# Hsa_circ_0097271 Knockdown Attenuates Osteosarcoma Progression via Regulating miR-640/MCAM Pathway

**DOI:** 10.1155/2022/8084034

**Published:** 2022-10-26

**Authors:** Peng Sun, Xuan Yang

**Affiliations:** Joint Surgery, Puren Hospital Affiliated to Wuhan University of Science and Technology, Wuhan, 430081 Hubei, China

## Abstract

**Background:**

The dysregulation of circular RNAs (circRNAs) participates in the malignant progression of multiple cancers, including osteosarcoma (OS). However, the role of circ_0097271 in OS development remains unclear. We thus aimed at unveiling the functional role and mechanism of circ_0097271 in OS.

**Methods:**

The expressions of circ_0097271, miR-640, and MCAM in OS were analyzed by qPCR. Cell proliferation and migration were inspected by CCK-8 assay, colony formation assay, and Transwell assay. Circ_0097271's role *in vivo* was assayed by establishing animal models. The predicted binding relationship between miR-640 and circ_0097271 or MCAM was verified by dual-luciferase reporter or RIP assay.

**Results:**

Circ_0097271's expression was enhanced in OS samples and cells. The knockdown of circ_0097271 restrained OS cell growth and migration, and its downregulation also blocked solid tumor growth *in vivo*. Circ_0097271 targeted miR-640 and negatively modulated miR-640 expression. MiR-640 was poorly expressed in OS, and its depletion recovered OS cell growth and migration that were repressed by circ_0097271 knockdown. MiR-640 bound to MCAM 3'UTR and thus suppressed MCAM expression. MCAM knockdown repressed OS cell growth and migration, while additional miR-640 depletion partially abolished the anticancer effects of MCAM knockdown in OS cells.

**Conclusion:**

Circ_0097271 is an oncogenic driver and contributes to OS development via targeting the miR-640/MCAM pathway, which provides a potential opinion for OS treatment.

## 1. Introduction

Osteosarcoma (OS) is the most common primary malignant bone tumor, most often occurring in adolescence [1]. Due to its highly aggressive biological behavior, amputation is considered the primary surgical treatment [2]. With a better understanding of the OS development and tremendous advances in treatment measures, most patients are treated with limb-preserving surgical resection combined with neoadjuvant chemotherapy whenever possible [2, 3]. Accordingly, 5-year survival rates have increased from no more than 30% to more than 70% [4, 5]. As molecular pathogenesis continues to advance, gene therapy has become a controllable, targeted, and specific treatment for OS [6]. Therefore, novel oncogenic factors should be identified to further provide treatment strategies for OS.

CircRNAs are a new class of regulatory factors that play specific roles in tumor progression and are classified as noncoding RNAs. CircRNAs originate from precursor mRNA via a “back-splicing” mechanism and featured by covalently closed structure and high stability in eukaryotic organisms [7, 8]. Growing studies unveil that circRNAs have huge potencies to mediate chemoresistance, tumor initiation, and aggressive progression [9]. For example, the circRNA expression profile exhibited the upregulation of circ_0001564 in OS, and circ_0001564 contributed to OS cell growth and survival via acting as miRNA sponges [10]. Circ_0081001 expression was enhanced in OS with methotrexate resistance, and depletion of circ_0081001 strengthened methotrexate chemosensitivity to repress OS development [11]. Unfortunately, numerous circRNAs have not been functionally exploited in OS. Circ_0097271 is derived from ATPase sarcoplasmic/endoplasmic reticulum Ca^2+^ transporting 2 (ATP2A2) genes by back-splicing. In view of the blank of circ_0097271' role in OS, we focus on circ_0097271 and investigate its functional role and mechanism in OS progression.

The bioinformatics tool circInteractome presents that miR-640 was a putative target of circ_0097271 because circ_0097271 sequence fragment contains miR-640 binding sites [12]. MiR-640 was shown as a tumor suppressor in breast cancer [13], whereas, the role of miR-640 in multiple cancers, including OS, remains unclear. The bioinformatics tool TargetScan displays that miR-640 has binding site on the 3'UTR of MCAM [14], implying that MCAM was a potential target gene of miR-640. MCAM, also known as CD146, is a cell surface adhesion molecule that is widely declaimed to facilitate cancer progression and metastasis [15]. MCAM dysregulation was also involved in OS development [16, 17]. However, the interplays between miR-640 and MCAM in OS development are unclear, and the upstream regulators of MCAM have not been fully identified.

Our present work for the first time investigated the expression pattern and functional role of circ_0097271 in OS development. In addition, we validated the binding relationship between miR-640 and circ_0097271 or MCAM in OS development to unveil the regulatory mechanism of circ_0097271. Our aim was to further understand OS pathogenesis from the insight of a novel circRNA, circ_0097271.

## 2. Materials and Methods

### 2.1. Human Samples

Human OS samples and matched noncancer normal control tissues were removed from 38 OS cases at our hospital. Before biopsy, all patients were firstly diagnosed with OS and had never received therapeutic measures against OS, such as chemotherapy and radiotherapy. Patients with other bone diseases or systemic diseases were excluded. The informed consent was acquired from each case. The procedures of human sample use were approved by our hospital.

### 2.2. Cell Culture and Cell Transfection

Human OS cells, Saos-2 (RRID: CVCL_0548) (Bena, Beijing, China), SW1353 (RRID: CVCL_0543) (Bena), SOSP-9607 (RRID: CVCL_4V80) (FenghBio, Changsha, China), and HOS (RRID: CVCL_0312) (Procell, Wuhan, China) were cultured in 90% DMEM (Sigma-Aldrich, USA) added with 10% FBS (Sigma-Aldrich) in an incubator (37°C, with 5% CO_2_).

We customized circ_0097271-specific or MCAM-specific small interference RNA (si-circ or si-MCAM) and their matched control (si-NC) from FenghBio. MiR-640 mimic, miR-NC, miR-640 inhibitor, and inhibitor-NC were directly bought from Ribobio (Guangzhou, China). When 70-80% cells were fused, the experimental cells were transfected with different oligonucleotides using Lipofectamine™ 2000 (11668019, Invitrogen, USA). 24 hours later, cells were collected and used for transfection efficiency detection. The sequence of siRNA is shown in Supplementary Table 1.

### 2.3. Quantitative Real-Time PCR (qPCR)

Total RNA was acquired from samples using the TRIzol reagent (15596-018, Invitrogen). Next, two commercial reverse transcription kits, including First-Strand cDNA Synthesis Kit (C0210A, GeneCopoeia, Shanghai, China) and miRNA First-Strand cDNA Synthesis Kit (QP013, GeneCopoeia), were utilized for cDNA synthesis. The diluted cDNA was reacted with SYBR Mixture (CW3008M, Cwbio, Beijing, China) for qPCR detection through a LightCycler 96 thermocycler (Roche, Switzerland). The expression of circ_0097271 and MCAM was normalized by GAPDH, and miR-640's expression was normalized by U6. The expression level was evaluated using the 2^−*ΔΔ*Ct^ method. We displayed primer sequences in Table 1.

### 2.4. RNase R Assay

Total RNA was acquired and experienced with RNase R (R0301, Geneseed, Guangzhou, China) for 15 minutes at 37°C. After treatment, total RNA was reverse-transcribed and used for qPCR as abovementioned.

### 2.5. Subcellular Location Assay

For circRNA subcellular location analysis, we briefly isolated cytoplasmic RNA and nuclear RNA from OS cells using the commercial PARIS kit (AM1921, Invitrogen) as the protocol suggested. The abundance of circ_0097271 in different locations was analyzed by qPCR assay, using GAPDH as an internal reference in the cytoplasm and U6 as an internal reference in the nucleus.

### 2.6. CCK-8 Assay

We prepared the transfected cells into 96-well plates at a density of 1500 cells/well in 100 *μ*L culture medium. Every 24 h interval (24, 48, 72, and 96 hours), CCK-8 (96992, Sigma-Aldrich) was added into each corresponding test well with 10 *μ*L per well, and cells were next incubated for additional 4 hours in incubators. The optical density (OD) was evaluated by measuring the 450 nm absorbance using a microplate reader (Biotek, USA).

### 2.7. Colony Formation Assay

We prepared the transfected cells into cell culture dishes at a density of 300 cells/well. Cells in complete culture medium were cultured in incubators for 12 days. The medium was refreshed every two days to induce colony formation. At last, cell debris was removed by PBS washing, and cell colonies (over 50 cells) after methanol fixation and 0.1% crystal violet (Sangon Biotech, Shanghai, China) staining were observed by light microscopy (Nikon, Japan).

### 2.8. Transwell Migration Assay

For migration analysis, 5 × 10^5^ cells were placed into the upper chamber (8-*μ*m; Corning, USA) and maintained in 500 *μ*L serum-free medium. The bottom of chambers was supplemented with matched culture medium (800 *μ*L) containing 20% FBS. After 24 h incubation, cells migrated to the low surface were subjected to methanol fixation and 0.1% crystal violet staining. Representative pictures were captured by light microscopy in random 5 fields.

### 2.9. Animal Study

Nude mice (female, 6-week-old) used in this study were bought from Vital River (Beijing, China) and then acclimated for one week in pathogen-free animal room. Circ_0097271-specific short-hairpin-RNA (sh-circ) and its matched control (sh-NC) were synthesized by Geneseed and packaged into lentiviral vectors. The lentiviral particles of sh-circ or sh-NC were used to infect Saos-2 cells to mediate circ_0097271 stable downregulation. To construct transplanted tumor models, the infected Saos-2 cells (2 × 10^6^ cells per mouse) were hypodermically injected into the groin of nude mice, with 5 mice in each group. Tumor volumes (length × width^2^ × 0.5) were weekly recorded by a vernier caliper. After 5 weeks, all mice were euthanized by intraperitoneal injection with pentobarbital. Then, tumor tissues were excised from animal body and used for further analysis. The procedures of animal use were approved by our hospital.

### 2.10. Dual-Luciferase Reporter Assay

According to the predicted multiple binding sites between circ_0097271 and miR-640, circ_0097271 reporter vectors, including wild-type (WT), mutation at binding site 1 (Mut1), mutation at binding site 2 (Mut2), and common mutation (Co-Mut), were all constructed into pmirGLO vector to verify these putative binding sites. Similarly, the WT and Mut reporter vectors of MCAM were also constructed. The WT/MUT reporter vector was transfected with miR-640 mimic or mimic-NC into Saos-2 and SW1353 cells using Lipofectamine™ 2000.48 hours later, we examined luciferase activities in line with the protocol of a dual luciferase reporter assay system (Promega, USA).

### 2.11. RIP Assay

Using a commercial RIP assay kit from Merck Millipore (17-704, USA), RIP assay was implemented to ensure the involvement of circ_0097271 with miR-640. In brief, Saos-2 and SW1353 cells were lysed, and cell lysates were incubated with magnetic beads coated with Ago2 antibody (Anti-Ago2) or IgG antibody (Anti-IgG). RNA complexes could be captured by antibody-coated beads and then eluted for qPCR analysis.

### 2.12. Western Blotting

Utilizing the RIPA Lysis Buffer (P0013E, Beyotime, Shanghai, China), total proteins were acquired from samples. Then, proteins were quantified using the BCA Protein Assay Kit (P0011, Beyotime). After denaturing by boiling for 10 minutes, equal amounts of protein lysates (40 *μ*g per lane) were separated by 10% SDS-PAGE and electron transferred to nitrocellulose membranes (Invitrogen). After blocking with 5% skim milk at room temperature for 1 hour, the membranes were probed with the primary antibody against MCAM (ab75769; 1/1000 dilution; Abcam, USA) or GAPDH (ab181602; 1/10000 dilution; Abcam) overnight at 4°C. After the incubation with the matched secondary antibody (ab205718; 1/20000 dilution; Abcam) for 2 hours at room temperature, the membranes were exposed to the ECL reagent (P0018AS, Beyotime) to display protein bands.

### 2.13. Statistical Analysis

GraphPad Prism 8 (GraphPad Software, USA) was used for figure drawing and statistical analysis of collected data from three independent experiments. Measurement data were displayed as mean ± standard deviation. Student's *t*-test was used for the comparison of the difference between two groups, and analysis of variance was used for the comparison among groups. The expression correlation between miR-640 and circ_0097271 or MCAM in OS samples was analyzed by Pearson's analysis. *P* < 0.05 was considered a statistically significant difference.

## 3. Results

### 3.1. Circ_0097271 Showed High Expression in OS Tumor Samples and Cells

**Figure 1:**
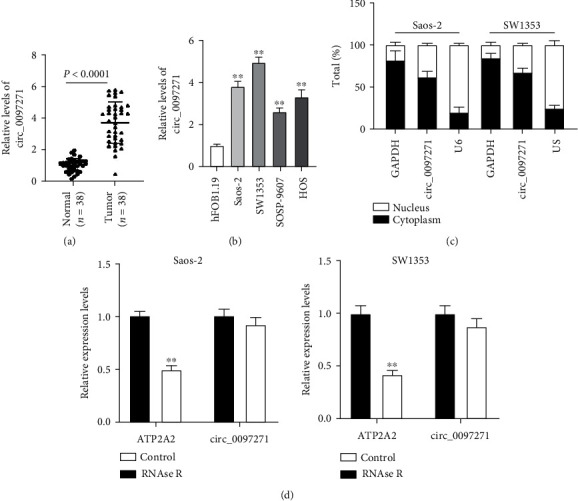
Circ_0097271 was highly expressed in OS. (a) Circ_0097271 expression in OS tumor samples and normal controls was investigated by qPCR. (b) Circ_0097271 expression in OS cells (Saos-2, SW1353, SOSP-9607, and HOS) and noncancer cells (hFOB1.19) was investigated by qPCR, ^∗∗^*P* < 0.01 relative to hFOB1.19. (c) Circ_0097271 expression in cytoplasm and nucleus of OS cells was checked by qPCR. (d) RNase R assay was applied to test the stability of circ_0097271, ^∗∗^*P* < 0.01 relative to Control.

To realize circ_0097271's expression pattern in OS, we conducted qPCR analysis and monitored that circ_0097271's expression was greatly reinforced in OS tumor samples in contrast to normal controls (Figure 1(a)). Diagnostic potentials of levels of circ_0097271 for OS were evaluated by ROC curve analysis with patients with OS tissues as true positive cases and normal tissues as true negative cases. Area under the curve was 0.9744, with standard error of 0.02340 and 95% confidence interval of 0.9285-1.020 (Supplementary Figure 1(a)). Circ_0097271's expression was also markedly enhanced in OS cell lines (Saos-2, SW1353, SOSP-9607, and HOS) in comparison to noncancer cell line (hFOB1.19) (Figure 1(b)). We selected Saos-2 and SW1353 cells in the following experiments because these two cell lines harbored relatively high expression of circ_0097271. Further analyses monitored that circ_0097271 was primarily distributed in the cytoplasmic fraction of Saos-2 and SW1353 cells but not in the nucleus (Figure 1(c)). Circ_0097271 was more resistant to RNase R digestion compared to its linear transcript, ATP2A2, because RNase R largely degraded ATP2A2 expression but rarely decreased circ_0097271 expression (Figure 1(d)). In summary, circ_0097271 was richly expressed in OS, with high stability.

### 3.2. Circ_0097271 Knockdown Exerted Antiproliferation and Antimigration Effects in OS Cells

**Figure 2:**
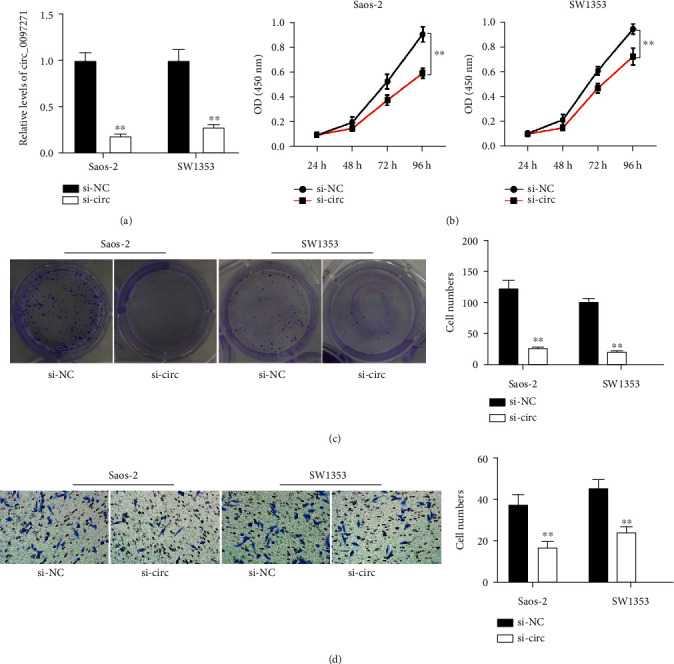
Circ_0097271 downregulation restrained OS cell growth and migration. (a) The inhibitory efficiency of si-circ on circ_0097271 expression was ensured by qPCR. (b) The effect of circ_0097271 downregulation on cell proliferation was evaluated by CCK-8 assay. (c) The effect of circ_0097271 downregulation on clone formation capacity was evaluated by colony formation assay. (d) The effect of circ_0097271 downregulation on cell migration was evaluated by Transwell assay. ^∗∗^*P* < 0.01 relative to si-NC.

We ensured that circ_0097271 expression was remarkably declined in si-circ-transfected Saos-2 and SW1353 cells in contrast to si-NC-transfected cells (Figure 2(a)). The OD450 value at 96 h of Saos-2 and SW1353 cells after si-circ transfection and the colony-forming ability of Saos-2 and SW1353 cells after si-circ transfection were greatly impaired, suggesting that circ_0097271 downregulation blocked OS cell growth (Figures 2(b) and 2(c)). In addition, we observed from Transwell assay that Saos-2 and SW1353 cells with circ_0097271 downregulation had the repressive migratory ability (Figure 2(d)). The data viewed that circ_0097271 knockdown restrained OS cell growth and migration.

### 3.3. Circ_0097271 Knockdown in Transplanted Tumor Models Restrained Tumor Growth

**Figure 3:**
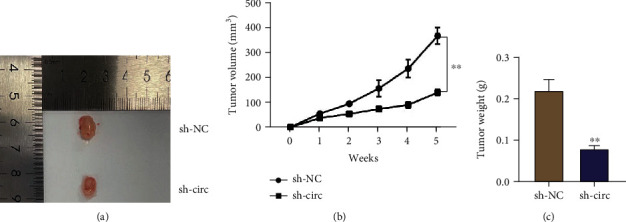
Circ_0097271 deficiency in animal models blocked tumor growth. (a) Representative images of tumor tissues from sh-circ and sh-NC-administered animal models. (b) Tumor volume was weekly measured during tumor growth. (c) Tumor weight was measured after tumor growth for 5 weeks. ^∗∗^*P* < 0.01 relative to sh-NC.

We constructed the transplanted tumor models by injecting Saos-2 cells (with sh-circ or sh-NC infection) into nude mice. Then, we observed that the infection of Saos-2 cells infected with sh-circ resulted in tumor tissues with smaller tumor size, while the infection of Saos-2 cells infected with sh-NC resulted in tumor tissues with bigger tumor size (Figure 3(a)). The detailed data showed that circ_0097271 downregulation in tumor tissues reduced tumor volumes and tumor weights (Figures 3(b) and 3(c)). Overall, circ_0097271 knockdown impeded tumor development in animal models.

### 3.4. MiR-640 Was Targeted by circ_0097271 and Was Downregulated in OS

**Figure 4:**
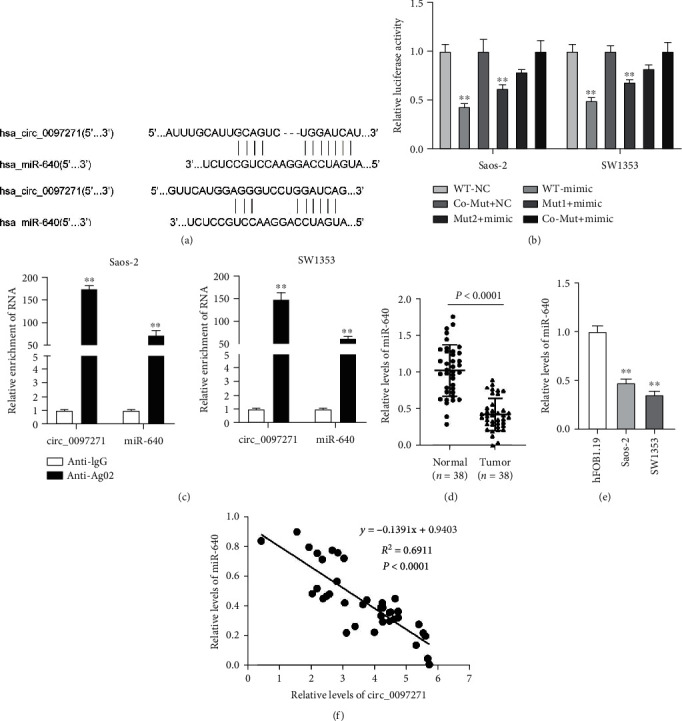
MiR-640 was a downstream target of circ_0097271. (a) The predicted binding sites between circ_0097271 and miR-640 were obtained from circInteractome tool. (b) The binding sites between circ_0097271 and miR-640 were then verified by dual-luciferase reporter assay, ^∗∗^*P* < 0.01 relative to WT-NC. (c) The binding between circ_0097271 and miR-640 was confirmed by RIP assay, ^∗∗^*P* < 0.01 relative to Anti-IgG. (d) MiR-640 expression in OS tumor tissues and normal controls was checked by qPCR. (e) MiR-640 expression in OS cells (Saos-2 and SW1353) and normal cells (hFOB1.19) was checked by qPCR, ^∗∗^*P* < 0.01 relative to hFOB1.19. (f) The linear relationship between miR-640 and circ_0097271 expression was analyzed by Pearson's analysis.

We exploited the downstream miRNAs targeted by circ_0097271, aiming to unveil circ_0097271's functional mechanism. Circ_0097271 was observed to have binding sites with miR-640 by circInteractome tool (Figure 4(a)). Then, we assembled multiple reporter vectors including WT or Mut sequences fragment of circ_0097271 to confirm the predicted binding sites between them. As a result, we found that miR-640 mimic largely lessened luciferase activities of WT reporter vector of circ_0097271 and partially reduced luciferase activities of Mut1 or Mut2 reporter vector of circ_0097271, while miR-640 mimic hardly weakened luciferase activities of Co-Mut reporter vector of circ_0097271 (Figure 4(b)), verifying that circ_0097271 had multiple binding sites with miR-640. Also, both circ_0097271 and miR-640 could be largely enriched by Anti-Ago2 in the RIP assay, in comparison to Anti-IgG, verifying the binding between circ_0097271 and miR-640 (Figure 4(c)). MiR-640 showed a low expression level in OS tumor samples in contrast to normal samples, as well as in OS cells (Saos-2 and SW1353) in contrast to hFOB1.19 cells (Figures 4(d) and 4(e)). Diagnostic potentials of levels of miR-640 for OS were evaluated by ROC curve analysis with patients with OS tissues as true positive cases and normal tissues as true negative cases. Area under the curve was 0.9204, with standard error of 0.03055 and 95% confidence interval of 0.8605-0.9803 (Supplementary Figure 1(b)). Moreover, miR-640 expression and circ_0097271 expression were inversely correlated in OS tumor samples (Figure 4(f)). In summary, miR-640 was targeted by circ_0097271 and showed the opposite expression pattern with circ_0097271 in OS.

### 3.5. The Antiproliferation and Antimigration Effects in OS Cells Caused by circ_0097271 Knockdown Were Overturned by miR-640 Depletion

**Figure 5:**
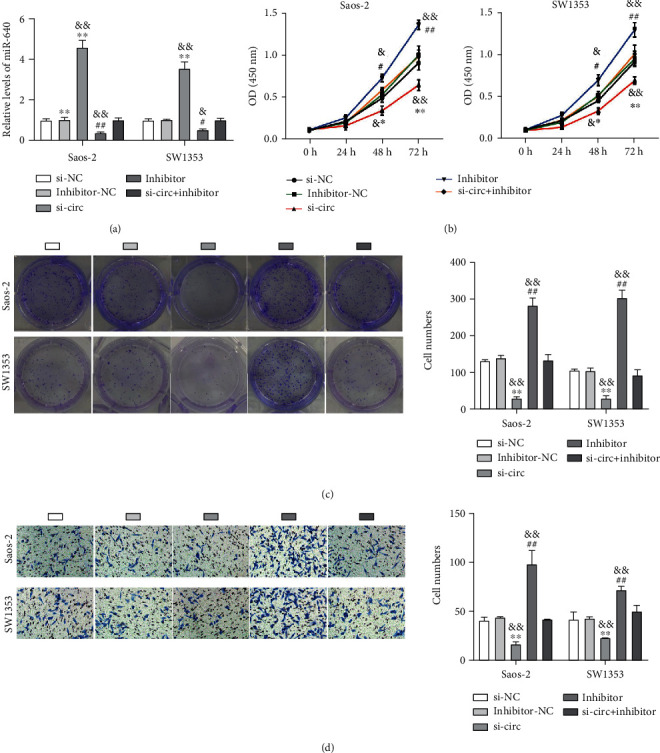
Circ_0097271 targeted miR-640 to promote OS cell malignant behaviors. Saos-2 and SW1353 cells were transfected with si-circ, si-NC, inhibitor, inhibitor-NC, or si-circ+inhibitor, and then used in the following experiments. (a) MiR-640 expression in these transfected cells was checked by qPCR. (b) The proliferation of these transfected cells was assessed by CCK-8 assay. (c) Clone formation capacity of these transfected cells was assessed by colony formation assay. (d) The migration of these transfected cells was assessed by Transwell assay. ^∗^*P* < 0.05, ^∗∗^*P* < 0.01 relative to si-NC; ^#^*P* < 0.05, ^##^*P* < 0.01 relative to inhibitor-NC; ^&^*P* < 0.05, ^&&^*P* < 0.01 relative to si-circ+inhibitor.

In view of the binding between circ_0097271 and miR-640, we further reduced miR-640 expression in circ_0097271-depleted OS cell to observe functional effects. At first, we ensured that miR-640 expression was greatly strengthened in Saos-2 and SW1353 cells transfected with si-circ but greatly declined in OS cells transfected with miR-640 inhibitor; In comparison to alone si-circ transfection, si-circ+inhibitor cotransfection partly impaired the expression of miR-640 (Figure 5(a)). In terms of function, inhibition of miR-640 largely strengthened the OD450 values of Saos-2 and SW1353 cells at 72 and 96 h posttransfection, enhanced OS cells' colony-forming ability, and cell migratory ability (Figures 5(b)–5(d)). Besides, these malignant cell behaviors suppressed by circ_0097271 knockdown, including cell viability, colony-forming ability, and migration, were all effectively restored by further miR-640 depletion (Figures 5(b)–5(d)). The data deemed that circ_0097271 inversely regulated miR-640 expression to promote OS cell development.

### 3.6. Mir-640 Negatively Modulated Its Downstream Target, MCAM

**Figure 6:**
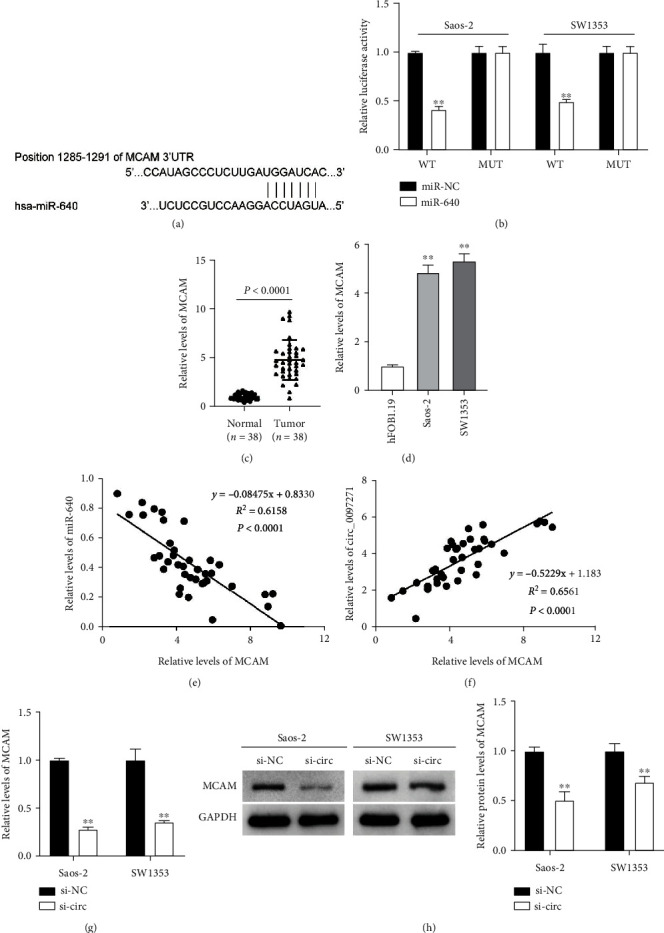
MCAM was a downstream target of miR-640. (a) The predicted binding site between miR-640 and MCAM 3'UTR was obtained from TargetScan. (b) The binding site between miR-640 and MCAM was verified by dual-luciferase reporter assay, ^∗∗^*P* < 0.01 relative to miR-NC. (c) MiR-640 expression in OS tumor tissues and normal controls was checked by qPCR. (d) MiR-640 expression in OS cells (Saos-2 and SW1353) and normal cells (hFOB1.19) was checked by qPCR, ^∗∗^*P* < 0.01 relative to hFOB1.19. (e) The linear relationship between miR-640 expression and MCAM expression in OS tissues was analyzed by Pearson's analysis. (f) The linear relationship between circ_0097271 expression and MCAM expression in OS tissues was analyzed by Pearson's analysis. (g) The expression of MCAM mRNA in si-circ or si-NC transfected Saos-2 and SW1353 cells was investigated by qPCR. ^∗∗^*P* < 0.01 relative to si-NC. (h) The expression of MCAM protein in si-circ or si-NC transfected Saos-2 and SW1353 cells was investigated by western blotting. ^∗∗^*P* < 0.01 relative to si-NC.

There are numerous potential functional genes targeted by miR-640. We thus utilized TargetScan tool to predict miR-640's target genes, and miR-640 was shown to possess binding site on MCAM 3′UTR (Figure 6(a)). MiR-640 mimic effectively diminished luciferase activities of WT reporter vector of MCAM but rarely changed luciferase activities of Mut reporter vector of MCAM, verifying this special binding site between miR-640 and MCAM 3′UTR (Figure 6(b)). MCAM expression was markedly reinforced in OS tumor samples in comparison to normal samples, as well as in OS cells (Saos-2 and SW1353) in comparison to hFOB1.19 cells (Figures 6(c) and 6(d)). Diagnostic potentials of levels of MCAM for OS were evaluated by ROC curve analysis with patients with OS tissues as true positive cases and normal tissues as true negative cases. Area under the curve was 0.9834, with standard error of 0.01543 and 95% confidence interval of 0.9531-1.014 (Supplementary Figure 1(c)). Additionally, we identified that MCAM expression was inversely linked to miR-640 expression, and positively linked to circ_0097271 expression in OS tumor samples (Figures 6(e) and 6(f)). Moreover, circ_0097271 knockdown inhibited MCAM mRNA and protein expression levels (Figures 6(g) and 6(h)). In summary, MCAM was a target of miR-640, and positively regulated by circ_0097271.

### 3.7. The Antiproliferation and Antimigration Effects in OS Cells Caused by MCAM Knockdown Were Abolished by miR-640 Depletion

**Figure 7:**
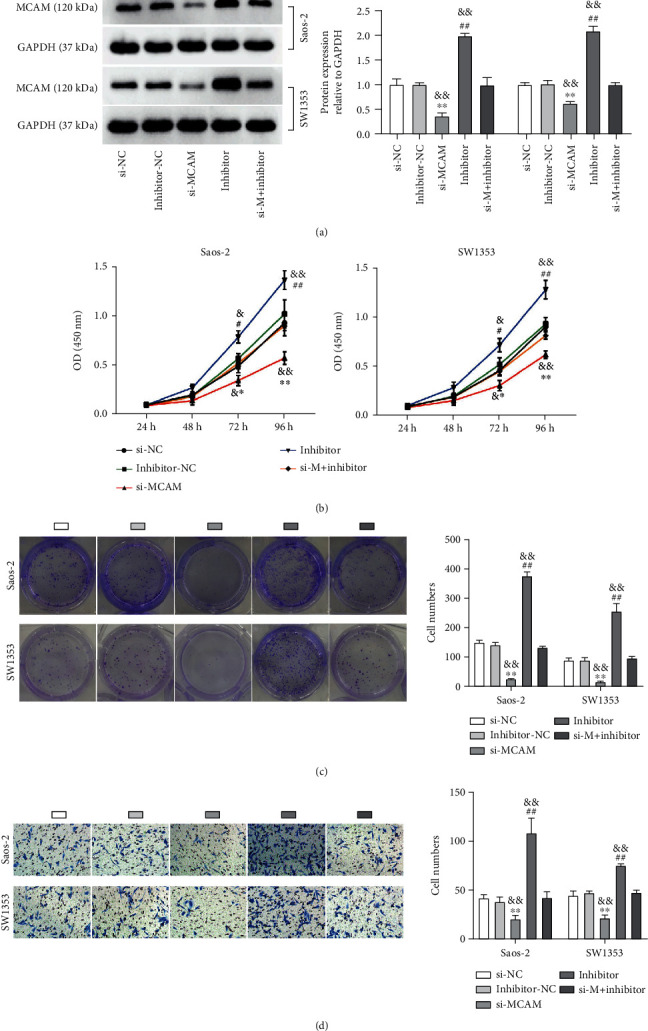
MiR-640 targeted MCAM to regulate OS cell functions. Saos-2 and SW1353 cells were introduced with si-MCAM, si-NC, inhibitor, inhibitor-NC, or si-MCAM+inhibitor. (a) The expression of MCAM protein in these transfected cells was investigated by western blotting. (b) The proliferation of these transfected cells was assessed by CCK-8 assay. (c) Clone formation capacity of these transfected cells was assessed by colony formation assay. (d) The migration of these transfected cells was evaluated by Transwell assay. ^∗^*P* < 0.05, ^∗∗^*P* < 0.01 relative to si-NC; ^#^*P* < 0.05, ^##^*P* < 0.01 relative to inhibitor-NC; ^&^*P* < 0.05, ^&&^*P* < 0.01 relative to si-M + inhibitor (si-M: si-MCAM).

We then performed rescue experiments to test the interactions between miR-640 and MCAM. At first, we observed that MCAM protein level was greatly reduced in Saos-2 and SW1353 cells transfected with si-MCAM but notably strengthened in OS cells transfected with miR-640 inhibitor; in comparison to alone si-MCAM transfection, si-MCAM+inhibitor cotransfection partially recovered MCAM expression (Figure 7(a)). In functional assays, MCAM downregulation strikingly weakened OS cell viability, colony-forming ability, and migratory capacity, which was completely opposite to the role of miR-640 depletion. Besides, MCAM downregulation-blocked cell viability, colony-forming ability, and migratory capacity were substantially restored by further miR-640 inhibition in OS cells (Figures 7(b)–7(d)). We concluded that miR-640 inhibition enhanced MCAM expression and thus attenuated the anti-cancer effects of MCAM knockdown.

## 4. Discussion

**Figure 8:**
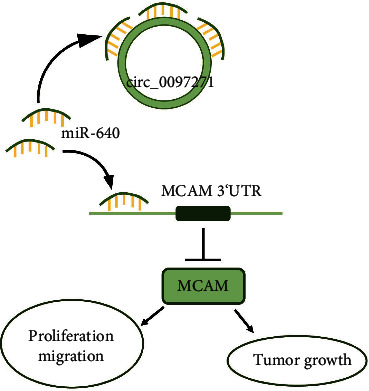
A flowchart of this study. Circ_0097271 is an oncogenic driver and contributes to OS development via targeting miR-640 and releasing MCAM.

Our study mainly discovered that circ_0097271's expression was greatly reinforced in OS samples and cells. Knockdown of circ_0097271 impeded OS cell growth and migration, and also repressed tumorigenesis in animal models. We further found that miR-640 was targeted by circ_0097271, and MCAM was a downstream target of circ_0097271/miR-640. Accordingly, we proposed that circ_0097271 accelerated OS malignant progression at least in part by modulating the miR-640/MCAM pathway (Figure 8).

CircRNAs exerting diverse functional effects in OS have been widely stated. For instance, circ_0001721 was overexpressed in OS, and circ_0001721 downregulation restrained OS development via repressing glycolysis metabolism, cell growth, migration, and invasion [18]. Circ_0078767 was also forcefully expressed in OS, and its ectopic expression largely aggravated OS cell growth, migratory ability and invasiveness and thus accelerated the growth of transplanted tumors [19]. Inversely, circ_0001105 was lowly expressed in OS and positively associated with survival rate of OS patients, and circ_0001105 overexpression largely restrained OS cell growth and invasion [20]. In view of the important and diverse role of circRNAs in OS, we characterized circ_0097271 and assayed its aberrant high expression in OS tissues and cells. Loss-of-function assays viewed that silencing circ_0097271 repressed OS cell growth and migration, and also hindered the development of transplanted tumors, hinting that circ_0097271 was a potential oncogenic driver in OS. Therefore, we speculated that circ_0097271 holds huge promise to be a therapeutic target for OS.

Regarding the regulatory mechanism of circRNAs, it is widely reported that circRNAs serve as competing endogenous RNAs (ceRNAs) to thus modulate the miRNA/mRNA networks [21]. For instance, circ_0001105 was shown to mediate OS proliferation and metastasis through miR-766-governed YTHDF2 axis [20], showing that circRNAs can function as miRNA sponges to regulate the expression of downstream mRNAs. Accordingly, we characterized the target miRNAs of circ_0001105 and discovered that circ_0097271 possessed binding sites with miR-640. MiR-640 was previously noted to be a suppressor in breast cancer, attributed to its inhibitory effects on Wnt7b/*β*-catenin oncogenic pathway [13]. Zhai et al. utilized meta-analysis to analyze microarray data in GEO database and discovered that miR-640 expression was markedly declined in hepatocellular carcinoma; besides, miR-640 enrichment restrained the proliferation of hepatocellular carcinoma cells [22]. Moreover, miR-640 overexpression could block Angiopoietin-1-induced endothelial cell angiogenesis, hinting that miR-640 had the potency to repress tumorigenesis [23], whereas, no studies reported the role of miR-640 in OS. We thus focused on miR-640 and identified its downregulation in OS tissues and cells. MiR-640 deficiency in OS cell aggravated cell growth and migration, and the suppression of circ_0097271 knockdown on OS cell growth and migration was partially attenuated by further miR-640 deficiency, suggesting that circ_0097271 targeted miR-640 to promote OS progression.

We further validated that MCAM was a target gene of miR-640. MCAM, widely known as CD146, has been regarded as an oncogenic biomarker and therapeutic target for multiple cancers [15, 24]. For instance, MCAM was highly regulated in breast cancer, and its downregulation was linked to the suppression of epithelial-mesenchymal transition and chemoresistance in breast cancer cells [25]. MCAM was strikingly elevated in small cell lung cancer with chemoresistance, and the suppression of MCAM largely repressed cancer cell proliferation and drug resistance [26]. MCAM was also mentioned to be overexpressed in OS, and its high expression was related to OS metastasis and poor prognosis [27, 28]. Overall, the oncogenic effect of MCAM in diverse cancers has been verified. Consistent with these findings, we displayed the high expression of MCAM in OS samples and cells. Knockdown of MCAM restrained OS cell growth and migration, while these anticancer effects caused by MCAM knockdown were largely abolished by miR-640 deficiency because miR-640 deficiency strengthened the expression level of MCAM in OS cells. MCAM was a functional molecule downstream of the circ_0097271/miR-640 pathway.

There are limitations in our present work. For example, the role of circ_0097271 on energy metabolism, cell cycle, and other cell behaviors is unclear, and more efforts should be done to address the detailed role of circ_0097271. Besides, the clinical practice and implication of circ_0097271 should be further summarized to enrich its potency as a biomarker for OS. These issues will be addressed in future work.

## 5. Conclusion

Circ_0097271 was overexpressed in OS and exerted oncogenic effects to enhance OS cell growth and migration. Circ_0097271 drove its oncogenic role in OS development at least in part by controlling the miR-640/MCAM axis. The management of circ_0097271-governed miR-640/MCAM axis might be a promising strategy for OS treatment.

## Figures and Tables

**Table 1:** Real-time PCR Primer synthesis list.

Gene	Sequences	
circ_0097271	Forward	5′-CTGTGGAAACCCTTGGTTGT-3′
	Reverse	5′-TCACCTGTGAGAATTGACTGG-3′

miR-640	Forward	5′-GCCCCTGCAGAGCACTGCGG-3′
	Reverse	5′-GGCCACCCGGCGGCCGGCAA-3′

MCAM	Forward	5′-CCGTCTCGTAAGAGCGAACT-3′
	Reverse	5′-CAGGGAAGGGAGCTGAAGTG-3′

U6	Forward	5′-CTCGCTTCGGCAGCACA-3′
	Reverse	5′-AACGCTTCACGAATTTGCGT-3′

GAPDH	Forward	5′-AGAAAAACCTGCCAAATATGATGAC-3′
	Reverse	5′-TGGGTGTCGCTGTTGAAGTC-3′

## Data Availability

All data generated or analyzed during this study are included in this article.

## Supplementary Materials

Supplementary 1 Supplementary Figure 1: tissues circ_0097271, miR-640, and MCAM were with diagnostic potentials for patients with OS ROC curve analysis. (a) The diagnostic potentials of circ_0097271. (b) The diagnostic potentials of miR-640. (c) The diagnostic potentials of MCAM.

Supplementary 2 Supplementary Table 1: the sequence of siRNA.
