# Supplementary figures and images for: Hsa_circ_0097271 Knockdown Attenuates Osteosarcoma Progression via Regulating miR-640/MCAM Pathway
Source: Dis Markers. 2022 Oct 26;2022:8084034. doi: 10.1155/2022/8084034 (PMC9630489; doi:10.1155/2022/8084034)

A

ROC curve: ROC of circ\_0097271

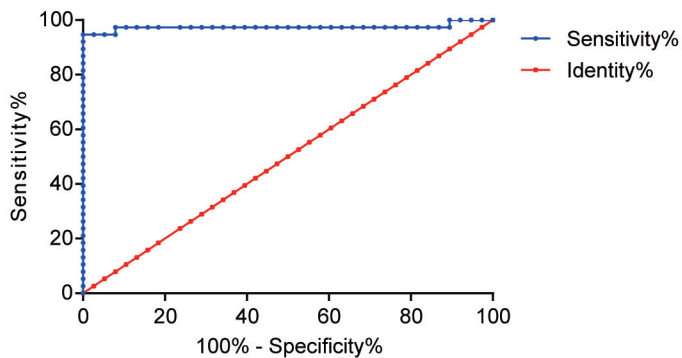

B

ROC curve: ROC of miR-640

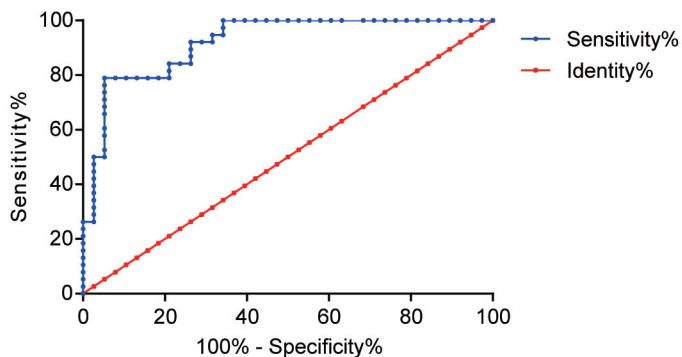

C

ROC curve: ROC of MCAM

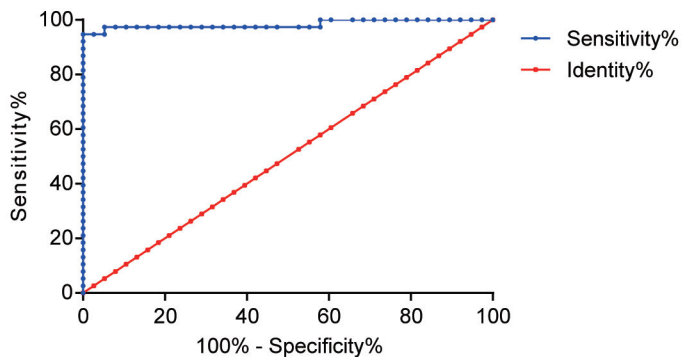

Supplement: Supplementary 1 — Supplementary Figure 1: tissues circ_0097271, miR-640, and MCAM were with diagnostic potentials for patients with OS ROC curve analysis. (a) The diagnostic potentials of circ_0097271. (b) The diagnostic potentials of miR-640. (c) The diagnostic potentials of MCAM. [file 8084034.f1.pdf]
