# Supplementary material for: Hsa_circ_0097271 Knockdown Attenuates Osteosarcoma Progression via Regulating miR-640/MCAM Pathway
Source: Dis Markers. 2022 Oct 26;2022:8084034. doi: 10.1155/2022/8084034 (PMC9630489; doi:10.1155/2022/8084034)
Supplement: Supplementary 2 — Supplementary Table 1: the sequence of siRNA. [file 8084034.f2.docx]

Supplementary Table 1 The sequence of siRNA

| Characteristic | Sequence (5’-3’) |
| --- | --- |
| si-circ | Sense: CUAAGAGUUGACCAGUCAAUU |
|  | Antisense: UUGACUGGUCAACUCUUAGUG |
| si-MCAM | Sense: GCUGGUUAAAGAAGACAAAGA |
|  | Antisense: UUUGUCUUCUUUAACCAGCUG |
| si-NC | Sense: UUCUCCGAACGUGUCACGUTT |
|  | Antisense: ACGUGACACGUUCGGAGAATT |
